# Epidemiology of Severe Acute Respiratory Illness and Risk Factors for Influenza Infection and Clinical Severity among Adults in Malawi, 2011–2013

**DOI:** 10.4269/ajtmh.17-0905

**Published:** 2018-07-23

**Authors:** Antonia Ho, Jane Mallewa, Ingrid Peterson, Miguel SanJoaquin, Shikha Garg, Naor Bar-Zeev, Mavis Menyere, Maaike Alaerts, Gugulethu Mapurisa, Moses Chilombe, Mulinda Nyirenda, David G. Lalloo, Camilla Rothe, Marc-Alain Widdowson, Meredith McMorrow, Neil French, Dean Everett, Robert S. Heyderman

**Affiliations:** 1 Malawi-Liverpool-Wellcome Trust Clinical Research Programme, College of Medicine, University of Malawi, Blantyre, Malawi;; 2 Institute of Infection and Global Health, University of Liverpool, Liverpool, United Kingdom;; 3 Department of Medicine, Queen Elizabeth Central Hospital, Blantyre, Malawi;; 4 World Bank, Phnom Penh, Cambodia;; 5 Influenza Division, Centers for Disease Control and Prevention, Atlanta, Georgia;; 6 Johns Hopkins Bloomberg School of Public Health, Baltimore, Maryland;; 7 Liverpool School of Tropical Medicine, Liverpool, United Kingdom;; 8 Division of Infectious Diseases and Tropical Medicine, Ludwig-Maximilians-University of Munich, Munich, Germany;; 9 Division of Global Health Protection, Centers for Disease Control and Prevention, Nairobi, Kenya;; 10 Division of Global Health Protection, Centers for Disease Control and Prevention, Atlanta, Georgia;; 11 Influenza Division, Centers for Disease Control and Prevention, Pretoria, South Africa;; 12 University of Edinburgh, Edinburgh, United Kingdom;; 13 Division of Infection and Immunity, University College London, London, United Kingdom

## Abstract

Data on the epidemiology of severe acute respiratory illness (SARI) in adults from low-income, high human immunodeficiency virus (HIV) prevalence African settings are scarce. We conducted adult SARI surveillance in Blantyre, Malawi. From January 2011 to December 2013, individuals aged ≥ 15 years with SARI (both inpatients and outpatients) were enrolled at a large teaching hospital in Blantyre, Malawi. Nasopharyngeal aspirates were tested for influenza and other respiratory viruses by polymerase chain reaction. We estimated hospital-attended influenza-positive SARI incidence rates and assessed factors associated with influenza positivity and clinical severity (Modified Early Warning Score > 4). We enrolled 1,126 SARI cases; 163 (14.5%) were positive for influenza. Human immunodeficiency virus prevalence was 50.3%. Annual incidence of hospital-attended influenza-associated SARI was 9.7–16.8 cases per 100,000 population. Human immunodeficiency virus was associated with a 5-fold greater incidence (incidence rate ratio 4.91, 95% confidence interval [CI]: 3.83–6.32). On multivariable analysis, female gender, as well as recruitment in hot, rainy season (December to March; adjusted odds ratios (aOR): 2.82, 95% CI: 1.57–5.06) and cool, dry season (April to August; aOR: 2.47, 95% CI: 1.35–4.15), was associated with influenza positivity, whereas influenza-positive patients were less likely to be HIV-infected (aOR: 0.59, 95% CI: 0.43–0.80) or have viral coinfection (aOR: 0.51, 95% CI: 0.36–0.73). Human immunodeficiency virus infection (aOR: 1.86; 95% CI: 1.35–2.56) and recruitment in hot, rainy season (aOR: 4.98, 95% CI: 3.17–7.81) were independently associated with clinical severity. In this high HIV prevalence population, influenza was associated with nearly 15% of hospital-attended SARI. Human immunodeficiency virus infection is an important risk factor for clinical severity in all-cause and influenza-associated SARI. Expanded access to HIV testing and antiretroviral treatment, as well as targeted influenza vaccination, may reduce the burden of SARI in Malawi and other high HIV prevalence settings.

## INTRODUCTION

Pneumonia is an important cause of morbidity and mortality in adults in sub-Saharan Africa.^1^ However, the burden of severe respiratory illness and the contribution of influenza and other respiratory viruses are not well documented in the region. Lack of diagnostic capacity, similarity of influenza presentation with common febrile illnesses such as malaria and bacterial pneumonia, and prioritization of other high-burden public health problems are likely contributory factors. A recent systematic review concluded that most of the sub-Saharan African countries had insufficient epidemiological data to develop rational strategies for influenza prevention and control.^2^ It is, therefore, unsurprising that although the World Health Organization (WHO) recommends seasonal influenza vaccine for high-risk groups, such as young children, pregnant women, and Human immunodeficiency virus (HIV)–infected individuals,^3^ few African countries have implemented these recommendations or have national policies.^4^

Following the 2009 influenza A(H1N1) pandemic, respiratory viral surveillance capacity has increased substantially in Africa.^5^ Currently, 23 sub-Saharan African countries contribute data to the WHO Global Surveillance and Response System.^6^ Emerging data suggest that influenza viruses are frequently detected in mild (6.7–40.4%) and severe (4.6–25.5%) acute respiratory presentations in the region^7^ and are associated with a higher mortality compared with developed settings because of the high prevalence of HIV infection and other comorbidities.^8^ However, only a handful of studies have focused on adults^9,10^ and few have comprehensively ascertained HIV status.

Malawi is a low-income country, ranked 170th of 188 countries in the Human Development Index.^11^ Active surveillance for influenza and other respiratory viruses was established at a large urban teaching hospital in Malawi in January 2011. In this high HIV prevalence and malaria-endemic setting, we aimed to describe the epidemiology and viral etiology and factors associated with clinical severity and influenza positivity among individuals aged ≥ 15 years with severe acute respiratory illness (SARI) during 2011–2013.

## MATERIALS AND METHODS

### Study site and setting.

Malawi has hot rainy (mean temperature > 22°C and rainfall > 100 mm; December to March), cool and dry (mean temperature < 22°C and rainfall < 50 mm; April to August), and hot and dry (mean temperature > 22°C and rainfall < 50 mm; September to November) seasons. The Queen Elizabeth Central Hospital (QECH) is the only government inpatient facility providing free health care to the 1.3 million residents of Blantyre District. Consequently, most individuals requiring hospitalization from this community will present to QECH. Human immunodeficiency virus prevalence in Blantyre is estimated at 17.7%,^12^ but up to 74% of patients admitted to the QECH medical wards are HIV infected.^13^ Malaria is endemic in Malawi (peak transmission months January to June), and malaria rapid diagnostic test (RDT) positivity is 8% among adult inpatients at QECH.^14^ Lower respiratory tract infections are the commonest cause of medical admission at QECH.^13^ There is no national influenza vaccination policy in Malawi. A WHO-led influenza A(H1N1)pdm09 vaccine campaign targeting health-care workers and pregnant women occurred in 2010.^15^

### Study procedures.

Patients aged 15 years and older presenting to the QECH Emergency Department during surveillance hours (8 am to 3 pm on weekdays) were screened for study eligibility. Consecutive patients from the start of the day fulfilling the SARI case definition were recruited (maximum four per day). Study staff collected demographic, clinical, and risk factor information using structured questionnaires and obtained nasopharyngeal aspirates and blood specimens for malaria and HIV testing.

SARI was defined as 1) an acute respiratory illness with symptom onset < 7 days, 2) reported or recorded fever (≥ 38°C), 3) cough or sore throat, and 4) shortness of breath or difficulty breathing. In our resource-limited setting, patients with severe illness requiring admission were often sent home. Therefore, hospital attendance (not admission) was required for study enrolment.

### Laboratory procedures.

The processing of respiratory specimens has been described previously.^16^ In brief, nasopharyngeal aspirates were stored at −80°C in Universal Transport Medium (Copan, Brescia, Italy). These were batch-tested for influenza A and B by real-time reverse transcription–polymerase chain reaction (rRT-PCR) using the CDC human influenza reverse transcription–PCR diagnostic panel (CDC Influenza Division, http://www.cdc.gov/ncird/flu.html). Influenza-positive specimens were subtyped using the CDC rRT-PCR protocol. The FTD respiratory pathogens 33 kit (Fast-track Diagnostics Ltd., Luxembourg, http://www.fast-trackdiagnostics.com) was used to detect coronaviruses OC43, NL63, HKU1, and 229E; parainfluenza viruses 1–4; respiratory syncytial viruses (RSV) A and B; enterovirus; human metapneumovirus; rhinovirus; adenovirus; and bocavirus. Samples with a Ct value < 40 were recorded as positive.

Human immunodeficiency virus testing (Alere Determine^™^ HIV-1/2, Waltham, MA, and Trinity Biotech Uni-Gold^™^ HIV, Bray, Co., Wicklow, Ireland) was performed according to WHO guidelines.^17^ Rapid diagnostic test for malaria (Paracheck Pf^®^, Orchid Biomedical Systems, Bamboli, Goa, India) was also performed in accordance with the manufacturer’s instructions.

### Climatic data.

Data on rainfall (millimeters), temperature (degree Celsius), and relative humidity (percentage) were obtained from the Malawi Department of Climate Change and Meteorological Services for 2011–2013.

### Statistical analysis.

Analysis was performed using Stata (Version 12.0; StataCorp Limited, College Station, TX). Monthly mean temperature, rainfall, and relative humidity were plotted against the number and proportion of influenza-positive SARI cases over the surveillance period to assess the association between climatic variations and influenza activity.

Numerators for minimum adult influenza-associated SARI incidence estimates were generated from the number of enrolled SARI with a positive influenza PCR that resided in the Blantyre district and adjusted for non-enrolment (during weekends and outside of surveillance hours on weekdays) by multiplying by the reciprocal of the proportion of recruited cases among all SARI cases attending the emergency department. The latter was recorded on the Surveillance Program of Inpatients and Epidemiology (SPINE) electronic data collection system.^13^ The annual incidence of hospital-attended influenza-positive SARI per 100,000 persons was estimated using the adjusted number of medically attended influenza-positive SARIs, divided by the census estimates of Blantyre District population aged ≥ 15 years for each year,^18^ and multiplied by 100,000. Incidence by HIV status was also calculated for individuals aged 15–49 years (in whom HIV prevalence is available^18^). Human immunodeficiency virus–associated incidence rate ratios (IRRs) were calculated by dividing the incidence in HIV-infected strata by the incidence in HIV-uninfected strata. 95% Confidence intervals (CIs) for incidence estimates and HIV-associated IRRs were calculated using the Poisson distribution.

Logistic regression was used to calculate odds ratios (OR) and 95% CIs to compare clinical variables between influenza-positive and influenza-negative patients. Multivariable logistic regression models were developed for two outcomes of interest: 1) influenza positivity and 2) clinical severity (defined as Modified Early Warning Score (MEWS) > 4).^19^ Modified early warning score is a simple physiological score based on five parameters (respiratory rate, heart rate, systolic blood pressure, temperature, and conscious level). It has been widely used in developed health-care settings to identify patients at risk of deterioration. A score of greater than 4 has been shown to be predictive of inpatient mortality in both well-resourced^19,20^ and African settings.^21,22^ Covariates with a *P* value of < 0.2 on univariable analysis, in addition to age, gender, and year of surveillance considered a priori confounders, were assessed for significance using backward stepwise selection. Odds ratios and 95% CIs were reported. Factors with 2-sided *P* values of < 0.05 were considered significant.

### Ethics approval.

Ethical approval for this study was obtained from the University of Malawi College of Medicine Research Ethics Committee (P.07/10/958), Liverpool School of Tropical Medicine (10.76), and the CDC through an ethical reliance. All participants provided written informed consent.

## RESULTS

### Demographic characteristics.

Between January 2011 and December 2013, 1,126 SARI cases aged 15 years and older were enrolled (Table 1). The median age was 33 years (interquartile range 26–42 years) and 489 (43.4%) were male. Of 1,109 patients with available HIV status (98.5%), 558 (50.3%) were HIV infected. Thirteen individuals reported receipt of influenza vaccination in the previous year.

**Table 1:** Characteristics of adult patients with SARI, Blantyre, Malawi, 2011–2013

Characteristic	SARI cases (*N* = 1,126) *n* (%)
Demographic characteristics	
Male	489 (43.4)
Age group (years)	
15–24	231 (20.5)
25–34	419 (37.2)
35–44	251 (22.3)
≥ 45	225 (20.0)
Underlying medical conditions	
HIV-positive*	558 (50.3)
Pregnant†	19/637 (3.0)
Current smoker	29 (2.8)
Antibiotics in the past 2 weeks	482 (46.5)
Reported influenza vaccination in the past year	13 (1.2)
Infectious agent identified	
Influenza virus (any type)	163 (14.5)
Influenza A	
H1N1pdm09	61 (37.4)
H3N2	47 (28.8)
Unsubtyped‡	1 (0.6)
Influenza B	50 (30.7)
Influenza A & B	3 (1.8)
Any virus detected§	533 (47.3)
≥ 2 viruses detected	154 (13.6)
Malaria RDT positive	28/911 (3.1)

HIV = human immunodeficiency virus; RDT = rapid diagnostic test; SARI = severe acute respiratory illness.

* HIV status–available for 1,109 patients.

† Pregnancy status established by self-report.

‡ Influenza A sample with cycle threshold values ≤ 40 that could not be subtyped.

§ Infection with at least one of influenza; adenovirus; bocavirus; coronavirus OC43, NL63, 229E and HKU1; enterovirus; human metapneumovirus; parainfluenza virus 1, 2, 3, and 4; rhinovirus, or respiratory syncytial virus.

### Viruses detected among SARI patients.

**Figure 1.**
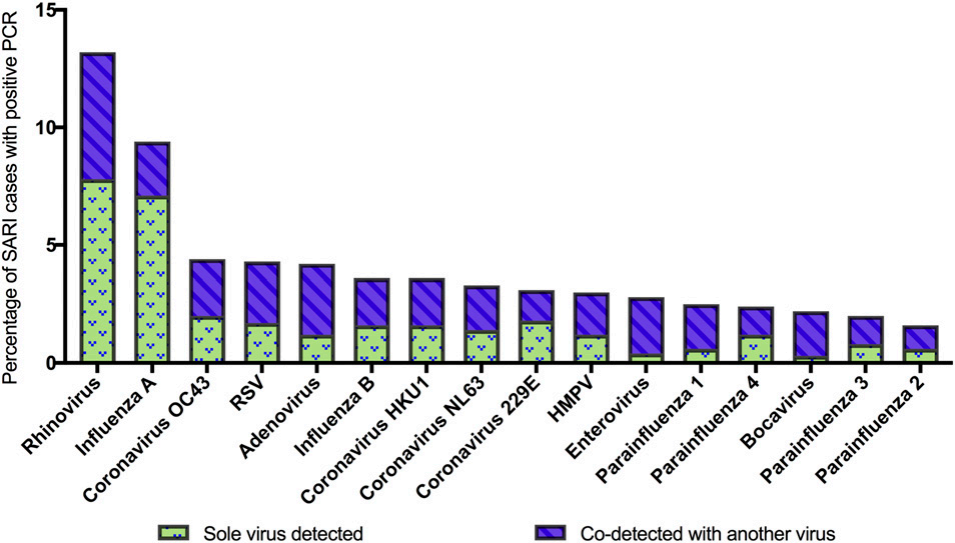
Respiratory viruses* detected in adults with severe acute respiratory illness (SARI), Blantyre, Malawi, 2011–2013. hMPV = human metapneumovirus; RSV = respiratory syncytial virus. *Respiratory viruses in the 33-pathogen multiplex polymerase chain reaction (PCR) include adenovirus, bocavirus, coronaviruses (OC43, NL63, 229E and HKU1), enterovirus, human metapneumovirus, parainfluenza viruses 1–4, respiratory syncytial virus, and rhinovirus. This figure appears in color at www.ajtmh.org.

One or more respiratory viruses were identified in 533 (47.3%) enrolled SARI cases (Table 1). Influenza viruses were detected in 163 (14.5%) SARI cases. When tested for the extended panel of respiratory viruses (*N* = 1,123) (Figure 1), rhinovirus was detected in 149 (13.3%), coronavirus OC43 in 49 (4.4%), RSV in 48 (4.2%), and adenovirus in 47 (4.2%). Influenza A and B were detected more frequently in HIV-uninfected than in HIV-infected SARI cases (influenza A, 12.0% versus 8.2%; influenza B 6.0% versus 3.1%; Supplemental Table 1), whereas the prevalence of other respiratory viruses did not differ by HIV status.

A single virus was isolated in 253 (22.5%) patients, whereas 154 (13.7%) individuals had two or more viruses detected. The highest proportion of viral co-detection was observed for bocavirus (21/24, 87.5%) and enterovirus (27/32; 84.4%), whereas the lowest proportion was observed in influenza A (26/108; 24.1%).

### Seasonality of influenza virus and malaria.

**Figure 2.**
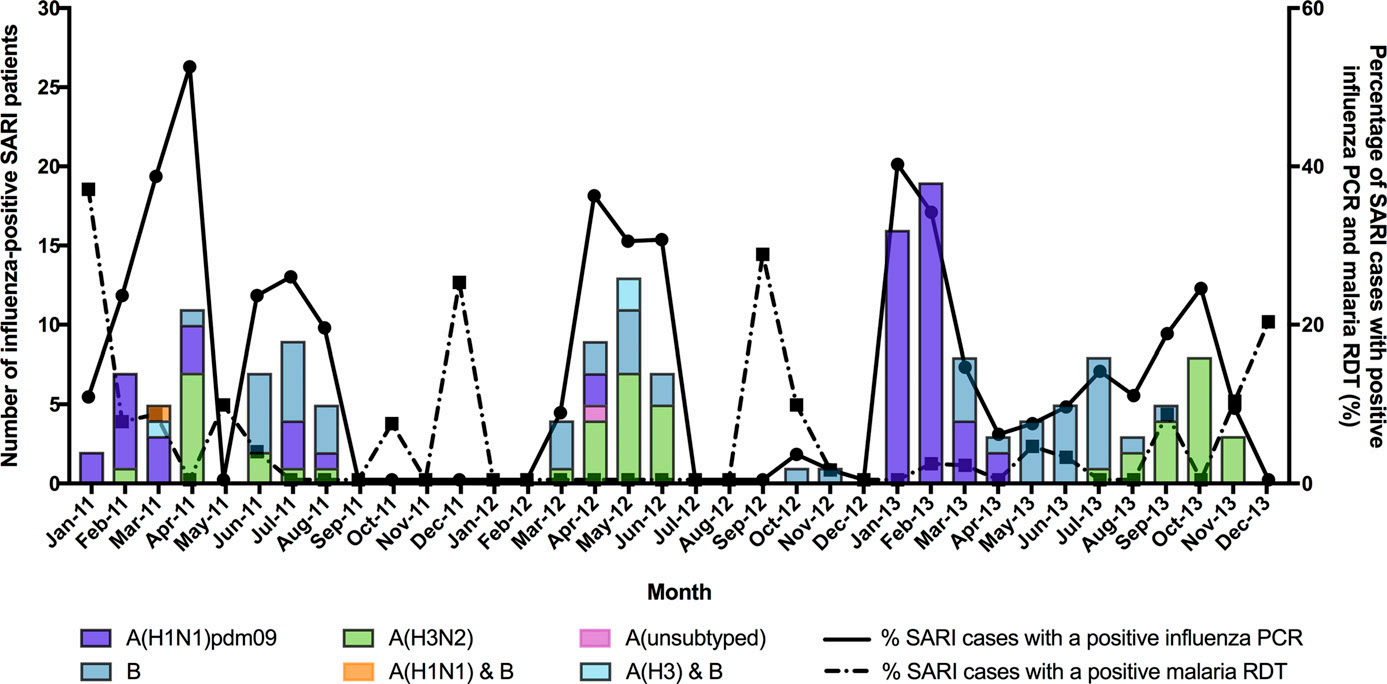
Seasonality of influenza for adults with severe acute respiratory illness (SARI), Blantyre, Malawi, 2011–2013. This figure appears in color at www.ajtmh.org.

Among the 163 influenza-positive SARI cases, 61 (37.2%) were influenza A(H1N1)pdm09, 47 (28.7%) were influenza A(H3N2), and 59 (30.5%) were influenza B. Three cases had influenza A and B coinfection and one influenza A sample was unsubtyped. Figure 2 illustrates the temporal distribution of influenza types and subtypes, as well as malaria RDT positivity. There were annual cycles of influenza activity, but timing of peak detection varied year to year. In 2011, influenza activity had a bimodal peak—in April and July. In 2012, influenza was detected between March and June only. By contrast, influenza was detected throughout 2013 but peaked in January and February. Peaks in influenza activity coincided with months with high relative humidity, but there was no correlation with rainfall or temperature (Supplemental Figure 1A–C). Influenza A(H1N1)pdm09, A(H3N2), and influenza B circulated in all 3 years; influenza A(H1N1)pdm09 was the predominant strain in 2011 (39.1%) and 2013 (49.4%), whereas influenza A(H3N2) was the most prevalent in 2012 (48.6%).

Of 911 SARI cases with available malaria RDT result, 28 (3.1%) were positive. There was no correlation between influenza and malaria activity (Figure 2). None of the cases with malaria were positive for influenza.

### Incidence estimates for hospital-attended influenza-positive SARIs.

The mean annual incidence of hospital-attended influenza-positive SARI per 100,000 for Blantyre residents aged 15 years and older was 14.4 per 100,000 (95% CI: 12.9–16.0) [16.8 (95% CI: 13.8–19.8) in 2011, 9.7 (95% CI: 7.4–11.9) in 2012, and 16.9 (95% CI: 140–19.8) in 2013].

Among individuals aged 15–49 years, the mean annual incidence of hospital-attended influenza-positive SARI in HIV-infected adults was 46.2 (95% CI: 37.5–56.3) per 100,000 and 9.4 (95% CI: 8.1–10.9) per 100,000 in HIV-uninfected adults (IRR: 4.92, 95% CI: 3.83–6.31).

### Factors associated with influenza positivity in SARI patients.

Compared with influenza-negative patients, a higher proportion of influenza-positive SARI patients reported headache (90.1 versus 83.6%, OR: 1.79, 95% CI: 1.02–3.13, *P* = 0.04). No other clinical feature differences by etiology were found (see Supplemental Table 2).

In the multivariable analysis (Table 2), female gender was associated with increased odds of influenza positivity compared with male gender (adjusted OR [aOR]: 1.57 (95% CI: 1.10–2.26). Greater influenza activity was observed in the hot, rainy season (17.5%; aOR: 2.82, 95% CI: 1.57–5.06) and the cold, dry season (16.5%; aOR: 2.37, 95% CI: 1.35–4.15), compared with the hot, dry season (6.7%). Furthermore, influenza positivity was inversely associated with HIV infection (aOR: 0.53, 95% CI: 0.36–0.76, *P* < 0.001) and co-detection with another respiratory virus (aOR: 0.46, 95% CI: 0.31–0.70, *P* < 0.001). Small numbers prohibited the evaluation of specific viral co-detection combinations with influenza.

**Table 2:** Factors associated with influenza PCR positivity in adults with SARI, Queen Elizabeth Central Hospital, Blantyre, Malawi, 2011–2013

Characteristic	Overall	Influenza virus negative, *N* (%)	Influenza virus positive, *N* (%)	Univariable*		Multivariable*†	
				OR (95% CI)	*P* value*	OR (95% CI)	*P* value*
Gendera							
Male	489	431 (88.1)	58 (11.9)	Ref	–	–	–
Female	636	532 (83.5)	105 (16.5)	1.47 (1.04–2.07)	0.03	1.57 (1.10–2.26)	0.01
Age group (years)							
15–24	231	191 (82.7)	40 (17.3)	1.37 (0.89–2.11)	0.36	1.23 (0.78–1.95)	0.68
25–34	419	359 (85.7)	60 (14.3)	1.10 (0.75–1.60)	–	1.06 (0.71–1.58)	–
≥ 35	476	413 (86.8)	63 (13.2)	Ref	–	Ref	–
Year of surveillance							
2011	251	205 (81.7)	46 (18.3)	2.12 (1.32–3.41)	–	2.85 (1.72–4.71)	–
2012	366	331 (90.4)	35 (9.6)	Ref	–	Ref	–
2013	509	427 (83.9)	82 (16.1)	1.82 (1.19–2.77)	0.003	1.84 (1.17–2.87)	< 0.001
Season of recruitment							
December–March (hot and rainy)	348	287 (82.5)	61 (17.5)	2.98 (1.71–5.17)	< 0.001	2.82 (1.57–5.06)	< 0.001
April–August (cool and dry)	508	424 (83.4)	84 (16.5)	2.77 (1.63–4.72)	–	2.37 (1.35–4.15)	–
September–November (hot and dry)	270	252 (93.3)	18 (6.7)	Ref	–	Ref	–
HIV status							
Negative	551	455 (82.6)	96 (17.4)	Ref	–	Ref	–
Positive	558	498 (82.3)	60 (10.8)	0.57 (0.40–0.81)	0.002	0.53 (0.36–0.76)	< 0.001
Medical history							
Malaria RDT—negative	883	745 (84.3)	138 (15.6)	–	–	–	–
Positive	28	28 (100)	0 (0)	–	–	–	–
Recent antibiotics—no	556	474 (85.2)	82 (14.8)	Ref	–	–	–
Yes	481	411 (85.4)	70 (14.6)	0.98 (0.70–1.39)	0.93	–	–
Current smoking—no	1,019	868 (85.1)	151 (14.8)	Ref	–	–	–
Yes	29	28 (96.5)	1 (3.5)	0.21 (0.03–1.52)	0.12	–	–
Co-detection with other respiratory virus(es)							
No	717	593 (82.7)	124 (17.3)	Ref	–	Ref	–
Yes	409	370 (90.5)	39 (9.5)	0.50 (0.34–0.74)	< 0.001	0.46 (0.31–0.70)	< 0.001

CI = confidence interval; HIV = human immunodeficiency virus; OR = odds ratio; PCR = polymerase chain reaction; RDT = rapid diagnostic test; SARI = severe acute respiratory illness.

* Logistic regression.

† Backward stepwise approach, including a priori confounders (age, gender, HIV status, and year of surveillance) and all variables with *P* < 0.20 in univariate analysis

### Factors associated with clinical severity.

We found that 238 of 1,126 patients with SARI (21.1%) had clinically severe disease (MEWS > 4). In multivariable analysis (Table 3), HIV infection was associated with a nearly 2-fold increase in clinical severity (aOR: 1.86, 95% CI: 1.35–2.56). SARI cases recruited in the hot, rainy season had five times increased odds of clinical severity, compared with those recruited in the hot, dry season (aOR: 4.98, 95% CI: 3.17–7.81). A higher proportion of clinically severe cases was also seen among cases recruited in 2011 (31.1 versus 17.9%, aOR: 2.31, 95% CI: 1.59–3.36, compared with cases recruited in 2013). Influenza infection was not associated with severe clinical presentation, nor were infection with other respiratory viruses, or viral coinfection.

**Table 3:** Factors associated with clinical severity (MEWS > 4) in adults with SARI, Blantyre, Malawi, 2011–2013

Characteristic	Number of cases with clinical severity *N* (%)	Univariable*		Multivariable*†	
		OR (95% CI)	*P* value	OR (95% CI)	*P* value
Gender					
Male	105/489 (21.5)	Ref	–	Ref	–
Female	133/636 (20.9)	0.97 (0.72–1.29)	0.82	0.93 (0.69–1.27)	0.65
Age group (years)					
15–24	46/231 (19.9)	Ref	–	Ref	–
25–34	87/419 (20.8)	1.05 (0.71–1.57)	–	0.82 (0.53–1.26)	–
≥ 35	105/476 (22.1)	1.11 (0.81–1.51)	0.80	0.96 (0.63–1.46)	0.55
Year of surveillance					
2011	78/251 (31.1)	2.07 (1.46–2.94)	0.001	2.31 (1.59–3.36)	< 0.001
2012	69/366 (18.9)	1.07 (0.75–1.51)	–	1.19 (0.82–1.72)	–
2013	91/509 (17.9)	Ref	–	Ref	–
Season					
December–March (hot and rainy)	125/348 (35.9)	4.32 (2.80–6.67)	–	4.98 (3.17–7.81)	< 0.001
April–August (cool and dry)	82/508 (16.1)	1.48 (0.95–2.31)	–	1.66 (1.05–2.63)	–
September–November (hot and dry)	31/270 (11.5)	Ref	–	Ref	–
HIV status					
Negative	91/551 (16.5)	Ref	–	Ref	–
Positive	143/558 (25.6)	1.74 (1.30–2.34)	< 0.001	1.86 (1.35–2.56)	< 0.001
Medical history					
Pregnancy–No	131/618 (21.2)	Ref	–	–	–
Yes	2/19 (10.5)	0.44 (0.10–1.92)	0.27	–	–
Recent antibiotics–No	134/548 (24.5)	Ref	–	–	–
Yes	101/483 (20.9)	0.82 (0.61–1.10)	0.19	–	–
Current smoker—no	224/946 (23.7)	Ref	–	–	–
Yes	14/102 (13.7)	0.25 (0.06–1.04)	0.06	–	–
Malaria RDT—Negative	213/883 (24.1)	Ref	–	–	–
Positive	6/28 (21.4)	0.86 (0.34–2.14)	0.74	–	–
Influenza—negative	198/962 (20.6)	Ref	–	–	–
Positive	40/163 (24.5)	1.25 (0.85–1.85)	0.25	–	–
Viral co-infections					
No	198/976 (20.3)	Ref	–	–	–
Yes	40/150 (26.7)	1.37 (0.93–2.03)	0.11	–	–
Other respiratory viruses					
Adenovirus—negative	228/1,076 (21.2)	Ref	–	–	–
Positive	10/47 (21.3)	1.01 (0.49–2.05)	0.99	–	–
Bocavirus—negative	232/1,099 (21.1)	Ref	–	–	–
Positive	6/24 (25.0)	1.25 (0.49–3.17)	0.65	–	–
Coronavirus					
OC43—negative	225/1,074 (21.0)	Ref	–	–	–
Positive	13/49 (26.5)	1.36 (0.71–2.61)	0.35	–	–
NL63—negative	227/1,086 (20.9)	Ref	–	–	–
Positive	11/37 (29.7)	1.60 (0.78–3.29)	0.20	–	–
229E—negative	227/1,088 (20.9)	Ref	–	–	–
Positive	11/35 (31.4)	1.74 (0.84–3.60)	0.14	–	–
HKU1—negative	220/1,063 (20.7)	Ref	–	–	–
Positive	7/40 (17.5)	0.81 (0.35–1.86)	0.62	–	–
Enterovirus—negative	236/1,091 (21.6)	Ref	–	–	–
Positive	2/32 (6.3)	0.24 (0.06–1.02)	0.05	–	–
hMPV—negative	224/1,089 (20.6)	Ref	–	–	–
Positive	14/34 (41.2)	2.70 (1.34–5.44)	0.005	–	–
Parainfluenza virus					
1—Negative	230/1,095 (21.0)	Ref	–	–	–
Positive	8/28 (28.6)	1.50 (0.65–3.46)	0.34	–	–
2—Negative	231/1,105 (20.9)	Ref	–	–	–
Positive	7/18 (38.9)	2.40 (0.92–6.28)	0.07	–	–
3—Negative	234/1,100 (21.3)	Ref	–	–	–
Positive	4/23 (17.4)	0.78 (0.26–2.31)	0.65	–	–
4—Negative	233/1,095 (21.3)	Ref	–	–	–
Positive	5/28 (17.9)	0.80 (0.30–2.14)	0.66	–	–
RSV—negative	226/1,075 (21.0)	Ref	–	–	–
Positive	12/47 (25.0)	1.25 (0.64–2.45)	0.51	–	–
Rhinovirus—negative	201/974 (20.6)	Ref	–	–	–
Positive	37/149 (24.8)	1.27 (0.85–1.90)	0.24	–	–

CI = confidence interval; HIV = human immunodeficiency virus; MEWS = modified early warning score; OR = odds ratio; RDT = rapid diagnostic test; RSV = respiratory syncytial virus; SARI = severe acute respiratory infection.

* Logistic regression.

† Backward stepwise approach, including a priori confounders (age, gender, HIV status, and year of surveillance) and all variables with *P* < 0.20 in univariate analysis.

Among the 163 influenza-positive SARI cases, 40 (24.5%) had a MEWS > 4. Those infected with influenza A(H1N1)pdm09 subtype were significantly associated with clinical severity (64.1%; aOR: 5.40, 95% CI: 1.88–15.53) compared with those infected with influenza B (20.5%; aOR: 1.55, 95% CI: 0.47–5.06) and influenza A(H3N2) (15.4%; baseline). Human immunodeficiency virus infection also predicted severity among influenza-positive SARI cases (38.3% versus 16.7%; aOR: 3.73, 95% CI: 1.65–8.41) (Supplemental Table 3).

## DISCUSSION

Comprehensive hospital-based sentinel surveillance in our high HIV prevalence, malaria-endemic African setting has identified influenza as an important contributor to SARI in adults, substantiating data from other African studies.^9,10^ In the immediate post-pandemic period, influenza A(H1N1)pdm09 was the predominant strain in Malawi in 2011 and 2013 and was associated with increased clinical severity compared with other subtypes. Influenza activity corresponded to months with higher relative humidity, but not with malaria activity. Among adults with SARI, female gender, in addition to recruitment in hot, rainy and cool, dry seasons, were associated with influenza positivity. Although HIV-infected adults with SARI were more likely to have an alternative etiology to influenza, HIV-infected adults aged 15–49 years had a 5-fold greater incidence of hospital-attended influenza-positive SARI compared with HIV-uninfected adults. Furthermore, HIV infection predicted clinical severity in all-cause SARI and influenza-associated SARI.

The estimated annual incidence of hospital-attended influenza-positive SARI ranged from 9.7 to 16.9 per 100,000 adult population, similar to that reported in rural Kenya (0.3/1,000)^23^ but substantially lower than estimates by another Kenyan study (2.8/100 for influenza A and 0.2/100 for influenza B^9^) and a South African study (71–260/100,000 [in HIV-infected persons] and 5–44/100,000 [in HIV-uninfected persons]).^24^ This wide variation could be due to geographical and seasonal differences in disease burden, but is also likely attributable to varying methodologies and case definitions, in addition to differing health-seeking behavior and thresholds for hospital admission. Furthermore, the latter two studies included children aged 5–14 years, a group that typically has higher rates of influenza infection.^9,24^ It is important to stress that our incidence estimates represent minimum estimates because our surveillance only detected persons accessing care at QECH. A small proportion of patients may have presented to a traditional healer or to one of the two private hospitals in Blantyre; SARI cases may not consider their symptoms severe enough to warrant care; they may be too ill or too poor to attend, or may have died before presentation.^25^

Human immunodeficiency virus infection, identified in more than 50% of adults with SARI, was the sole individual risk factor associated with increased clinical severity. Indeed, several African adult pneumonia cohorts have reported a high prevalence of HIV infection (52–94%).^26–28^ Our result supports findings from others that HIV is an important driver of severe respiratory infection,^29^ including influenza, in sub-Saharan Africa.

Influenza was less commonly identified in HIV-infected compared with HIV-uninfected SARI cases. This likely reflects the different spectra of organisms affecting HIV-infected adults, with greater relative contribution of opportunistic pathogens such as *Mycobacterium tuberculosis*, *Pneumocystis jirovecii*, and *Streptococcus pneumoniae*, rather than a lower absolute risk. This has also been described in HIV-infected children^30^ and adults^10^ in South Africa. In fact, after taking into account population denominators, HIV-infected adults aged 15–49 years had a 5-fold greater incidence of influenza-positive SARI than HIV-uninfected adults. Having comprehensively ascertained HIV status, our study corroborates with studies in Malawi, Kenya, and South Africa that have identified HIV as a major risk factor for influenza burden^25^ and severe disease.^24,31,32^ These results suggest that early HIV testing and expanded access to antiretroviral treatment, in addition to targeted influenza vaccination, could potentially have a substantial impact on burden of SARI in urban Blantyre and other similar high HIV prevalence settings. Annual influenza vaccination is recommended for HIV-infected individuals.^3^ Influenza vaccination in HIV-infected adults is safe and effective (pooled efficacy 85%),^33^ but influenza vaccines are currently unavailable in most African countries.^4,34^

SARI cases recruited in hot, rainy season were associated with a 5-fold increased odds of clinical severity, compared with those recruited in the hot, dry season. This was also observed in our pediatric surveillance.^16^ The reason for this is unclear but could be related to other unmeasured infections (we were unable to determine the presence of bacterial pathogens in our SARI cases), seasonal patterns of health-care utilization, and seasonal malnutrition. The hot, rainy season in Malawi coincides with the “lean” season before harvest.^35^ A recent case-control study in Malawi identified food insecurity as a risk factor for influenza severity,^31^ thus supporting our latter hypothesis.

We identified at least one respiratory virus in nearly half of all SARI cases, higher than that described in South African adults,^10^ and in developed settings.^36,37^ Viral coinfections were common, occurring in 14% of adult SARI cases. We also found a nonsignificant trend toward increased severity in adults with viral coinfection (26.7% versus 20.3% with MEWS > 4, *P* = 0.11). There is growing recognition that viruses other than influenza, such as rhinovirus, adenovirus, hMPV, and parainfluenza viruses, can cause clinically severe disease. However, whereas the detection of influenza, RSV, and hMPV in adults with SARI likely indicates an etiologic role,^38,39^ the presence of other respiratory viruses is of uncertain significance, particularly as we did not enroll accompanying controls. Further understanding of the interactions and contribution of these viruses to severe respiratory disease will help to narrow the focus on pertinent targets for vaccine and antiviral development.

Our study has a number of limitations. First, we conducted single-site hospital-based surveillance. Although there are no other large inpatient facilities in Blantyre, we have not sampled from elsewhere in Malawi. Second, limiting recruitment to the first four cases of the day could have resulted in selection bias because individuals who present to hospital at different times of the day may have varying characteristics, such as health-seeking behavior or distance of residence from hospital. Third as discussed earlier, patients with SARI could have sought health care in facilities other than QECH, leading to an underestimation of our influenza-associated SARI rates. Under-ascertainment of SARI cases and resultant underestimation of incidence were also possible if SARI cases were not systematically recorded onto SPINE. Fourth, although we had near-complete ascertainment of HIV status (98.5%), data on CD4^+^ cell count and antiretroviral treatment status were not available. Comorbidities were also poorly recorded; thus, we were unable to evaluate chronic lung disease as a potential risk factor for influenza or adjust for underlying comorbidities in the multivariable analysis for clinical severity. Last, data on hospitalization and mortality were not systematically captured. Instead, we used the MEWS score as a surrogate marker for clinical severity. The score has been widely used in developed health-care settings to identify patients at risk of deterioration, and a threshold of greater than four is predictive of inpatient mortality.^19^ It has also been validated in other African settings.^21,22^

This study provides a baseline for understanding the complexities of SARI epidemiology in adults in Malawi and other similar settings. In this high HIV prevalence setting, respiratory viruses were commonly identified in adults with SARI and influenza has a prominent etiological role. Human immunodeficiency virus–infected adults are at particular risk of severe disease and have a higher burden of influenza-associated SARI than HIV-uninfected individuals. Ongoing surveillance for influenza and other respiratory viruses, with specific focus on severe disease in high-risk groups such as HIV-infected individuals and pregnant women, and greater effort to capture outcome data are critical to further characterize disease burden in these high-risk groups to inform public policy decisions. Improved HIV testing and early ART initiation, as well as targeted influenza vaccination could potentially substantially reduce the burden of SARI in Malawi and other sub-Saharan African countries with high HIV prevalence.

## Supplementary Material

Supplemental figures and tables
