# Supplementary material for: Epidemiology of Severe Acute Respiratory Illness and Risk Factors for Influenza Infection and Clinical Severity among Adults in Malawi, 2011–2013
Source: Am J Trop Med Hyg. 2018 Jul 23;99(3):772–9. doi: 10.4269/ajtmh.17-0905 (PMC6169174; doi:10.4269/ajtmh.17-0905)
Supplement: Supplementary file 1 [file tpmd170905.SD1.docx]

**Supplementary Table 1. Prevalence of respiratory viruses by HIV status in Adults with Severe Acute Respiratory Illness, Queen Elizabeth Central Hospital, Blantyre, Malawi, 2011 – 2013.**

| **Characteristic** | **Number of cases (%)** | | **P-value*** |  |
| --- | --- | --- | --- | --- |
|  | **HIV-positive**  **(n=556)** | **HIV-negative**  **(n=550)** |  |  |
| Adenovirus | 23 (4.1) | 22 (5.0) | 0.91 |  |
| Bocavirus | 14 (2.5) | 10 (1.8) | 0.42 |  |
| Coronavirus OC43 | 31 (5.6) | 18 (3.3) | 0.06 |  |
| Coronavirus NL63 | 21 (3.8) | 14 (2.6) | 0.24 |  |
| Coronavirus 229E | 15 (2.7) | 20 (3.6) | 0.37 |  |
| Coronavirus HKU1 | 15/542 (2.8) | 22/544 (4.0) | 0.25 |  |
| Enterovirus | 12 (2.2) | 19 (3.5) | 0.19 |  |
| Human metapneumovirus | 22 (4.0) | 12 (2.2) | 0.09 |  |
| Influenza A | 46 (8.2) | 66 (12.0) | 0.04 |  |
| Influenza B | 17 (3.1) | 33 (6.0) | 0.02 |  |
| Parainfluenza virus 1 | 10 (1.8) | 18 (3.3) | 0.12 |  |
| Parainfluenza virus 2 | 10 (1.8) | 8 (1.5) | 0.65 |  |
| Parainfluenza virus 3 | 12 (2.2) | 10 (1.8) | 0.69 |  |
| Parainfluenza virus 4 | 8 (1.4) | 15 (2.7) | 0.13 |  |
| Respiratory syncytial virus | 28 (5.0) | 18 (3.3) | 0.14 |  |
| Rhinovirus | 68 (12.2) | 80 (14.6) | 0.26 |  |
| Abbreviations: HIV, human immunodeficiency virus. | | | | |

**Supplementary Table 2. Clinical Characteristics by Influenza PCR Status in Adults with Severe Acute Respiratory Illness, Queen Elizabeth Central Hospital, Blantyre, Malawi, 2011 – 2013.**

| **Characteristic** |  | **Number of cases (%) with characteristic** | | **OR (95% CI)** | **P-value*** |
| --- | --- | --- | --- | --- | --- |
|  | **All SARI cases**  **(n=1126)** | **Influenza PCR positive**  **(n=163)** | **Influenza PCR negative**  **(n=963)** |  |  |
| **Symptoms** |  |  |  |  |  |
| **Fever >38°c** | 909/1120 (81.2) | **138/162 (85.2)** | **771/958 (80.5)** | **1.39 (0.88-2.23)** | **0.16** |
| **Cough** | 1016/1045 (97.2) | **149/152 (98.0)** | **867/893 (97.1)** | **1.49 (0.45-4.99)** | **0.52** |
| **Sore throat** | 687/1041 (66.0) | **95/150 (63.3)** | **592/891 (66.5)** | **0.87 (0.61-1.25)** | **0.45** |
| **Headache** | 884/1046 (84.5) | **137/152 (90.1)** | **747/894 (83.6)** | **1.79 (1.02-3.13)** | **0.04** |
| **Rhinorrhoea** | 762/1043 (73.1) | **113/152 (74.3)** | **649/891 (72.8)** | **1.08 (0.72-1.60)** | **0.03** |
| **Myalgia** | 884/1044 (84.6) | **129/152 (84.9)** | **755/892 (84.6)** | **1.02 (0.63-1.64)** | **0.94** |
| **Vomiting/diarrhoea** | 166/1044 (15.9) | **19/151 (12.6)** | **147/892 (16.5)** | **0.73 (0.44-1.22)** | **0.23** |
| **Signs** |  |  |  |  |  |
| **Confused** | 21/1043 (2.0) | **4/152 (2.6)** | **17/891 (1.9)** | **1.39 (0.46-4.19)** | **0.56** |
| **Systolic BP <90mmHg** | 140/1043 (13.4) | **26/159 (17.5)** | **114/894 (12.8)** | **1.44 (0.91-2.30)** | **0.12** |
| **Heart rate >120/minute** | 407/1044 (39.0) | **66/150 (44.0)** | **341/894 (38.1)** | **1.27 (0.90-1.80)** | **0.18** |
| **Respiratory rate >30/minute** | 479/1037 (46.1) | **75/148 (50.3)** | **404/889 (45.4)** | **1.23 (0.87-1.74)** | **0.24** |
| **Oxygen saturation <90% on air** | 124/1048 (11.8) | **21/150 (14.0)** | **103/898 (11.5)** | **1.25 (0.76-2.08)** | **0.37** |
| **Abbreviations: PCR=polymerase chain reaction; OR, odds ratio; CI, confidence interval; BP, blood pressure** | | | | | |

**Supplementary Table 3. Factors associated with clinical severity (MEWS > 4) in adults with influenza-associated severe acute respiratory illness (SARI), Blantyre, Malawi, 2011–2013.**

| **Characteristic** | **Number of cases with clinical severity (n=40) (%)** | **Univariable^a^** | |  | **Multivariable^a,b^** | |
| --- | --- | --- | --- | --- | --- | --- |
|  |  | **OR (95% CI)** | **P-value** |  | **OR (95% CI)** | **P-value** |
| **Sex** |  |  |  |  |  |  |
| Male | 10/58 (17.2) | Ref |  |  |  |  |
| Female | 30/106 (28.6) | 1.92 (0.86-4.28) | 0.11 |  |  |  |
| **Age group (years)** |  |  |  |  |  |  |
| 15-24 | 7/40 (17.5) | Ref |  |  |  |  |
| 25-34 | 12/60 (20.0) | 1.18 (0.42-3.31) |  |  |  |  |
| > 35 | 21/63 (33.0) | 2.36 (0.89-6.21) | 0.12 |  |  |  |
| **Year of surveillance** |  |  |  |  |  |  |
| 2011 | 15/46 (32.6) | 2.90 (0.93-8.99) |  |  |  |  |
| 2012 | 5/35 (14.3) | Ref |  |  |  |  |
| 2013 | 20/82 (24.4) | 1.94 (0.66-5.66) | 0.15 |  |  |  |
| **Season** |  |  |  |  |  |  |
| Hot and rainy | 27/61 (44.3) | 13.50 (1.69-106.96) | <0.001 |  |  |  |
| Cold and dry | 12/83 (14.2) | 2.83 (0.34-23.30) |  |  |  |  |
| Hot and dry | 1/18 (5.6) | Ref |  |  |  |  |
| **HIV status** |  |  |  |  |  |  |
| Negative | 16/96 (16.7) | Ref |  |  | Ref |  |
| Positive | 23/60 (38.3) | 3.11 (1.47-6.56) | 0.003 |  | 3.72 (1.65-8.42) | 0.002 |
| **Medical history** |  |  |  |  |  |  |
| Pregnancy - No | 39/157 (24.8) | Ref |  |  |  |  |
| - Yes | 1/6 (16.7) | 0.61 (0.07-5.34) | 0.65 |  |  |  |
| Recent antibiotics – No | 21/82 (25.6) | Ref |  |  |  |  |
| – Yes | 19/70 (27.1) | 1.08 (.53-2.23) | 0.83 |  |  |  |
| **Influenza subtype** |  |  |  |  |  |  |
| A(pdm)09 | 25/61 (41.0) | 4.75 (1.75-12.86) |  |  | 5.40 (1.88-15.53) |  |
| A(H3N2) | 6/47 (12.8) | Ref |  |  | Ref |  |
| B | 8/50 (16.0) | 1.30 (.042-4.08) | <0.001 |  | 1.55 (0.47-5.06) | <0.001 |
| **Co-detection with other respiratory virus(es)** |  |  |  |  |  |  |
| No | 30/124 (24.2) | Ref |  |  |  |  |
| Yes | 10/39 (25.6) | 1.08 (0.47-2.47) | 0.86 |  |  |  |

OR – odds ratio, CI – confidence interval, HIV – human immunodeficiency virus.

^a^Logistic regression.

^b^Backward stepwise approach, including all variables with p<0.20 in univariate analysis.
