# Supplementary figures and images for: Epidemiology of Severe Acute Respiratory Illness and Risk Factors for Influenza Infection and Clinical Severity among Adults in Malawi, 2011–2013
Source: Am J Trop Med Hyg. 2018 Jul 23;99(3):772–9. doi: 10.4269/ajtmh.17-0905 (PMC6169174; doi:10.4269/ajtmh.17-0905)

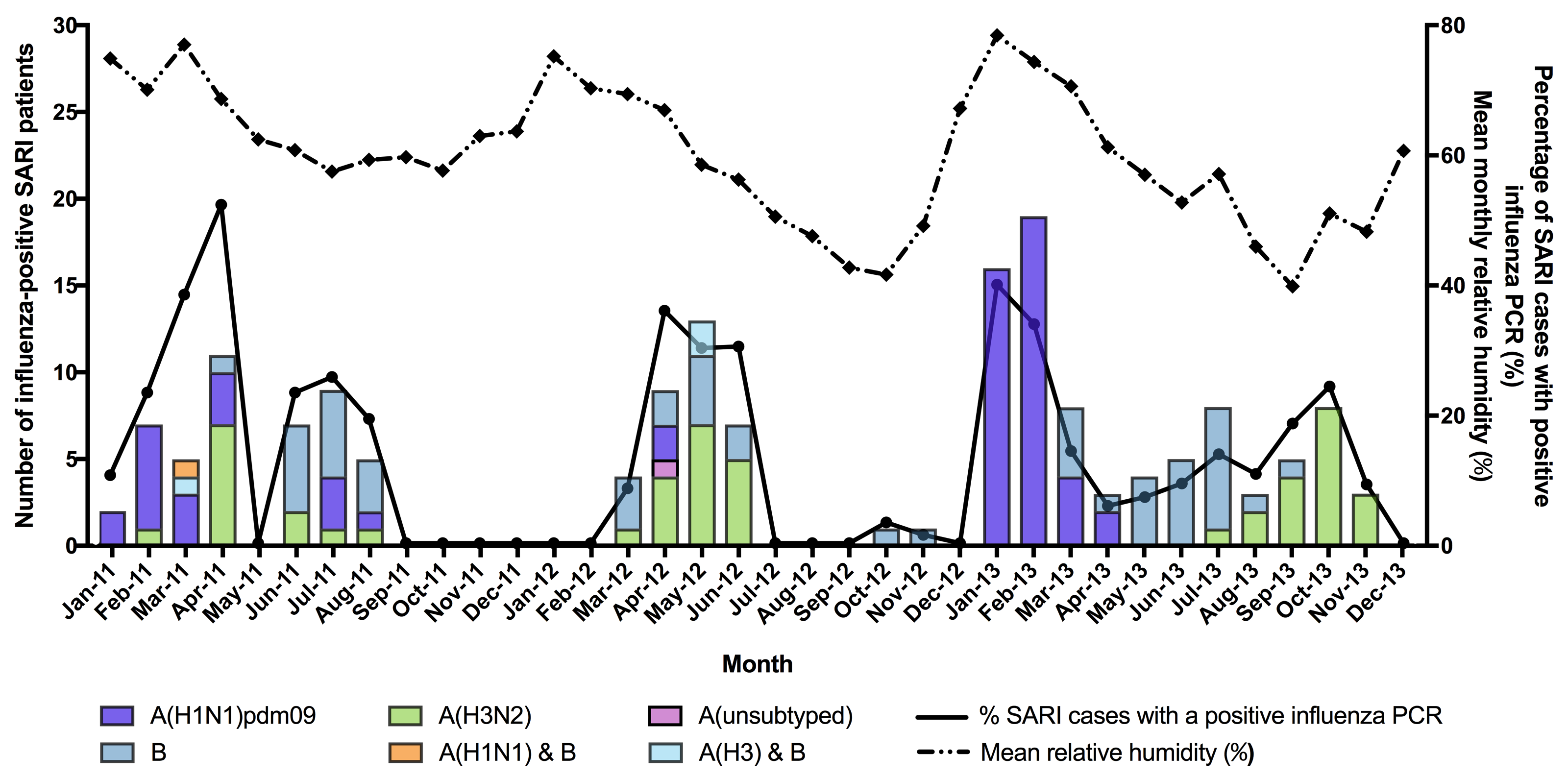

Supplement: Supplementary file 2 [file tpmd170905.SD2.tiff]

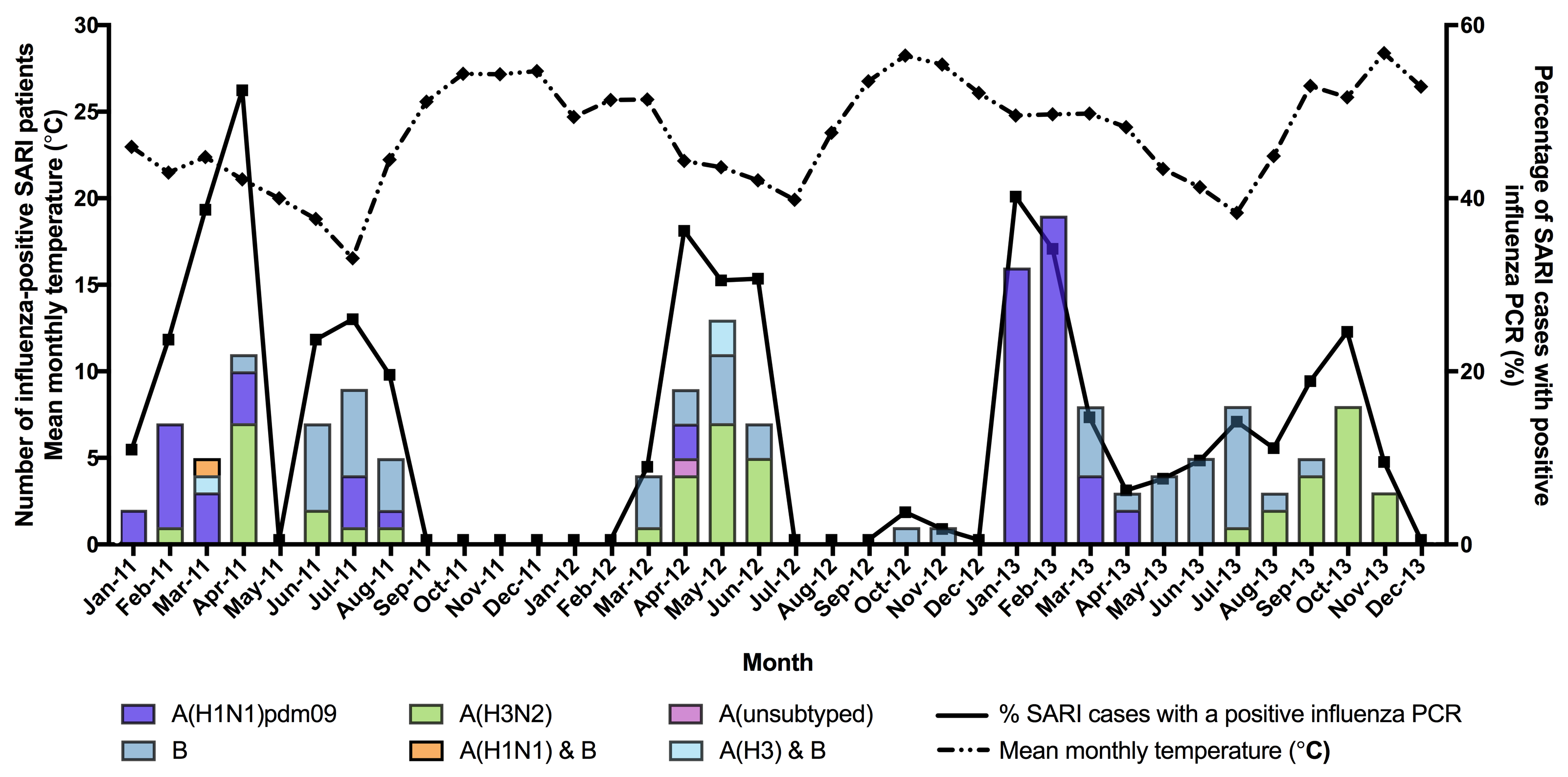

Supplement: Supplementary file 3 [file tpmd170905.SD3.tiff]

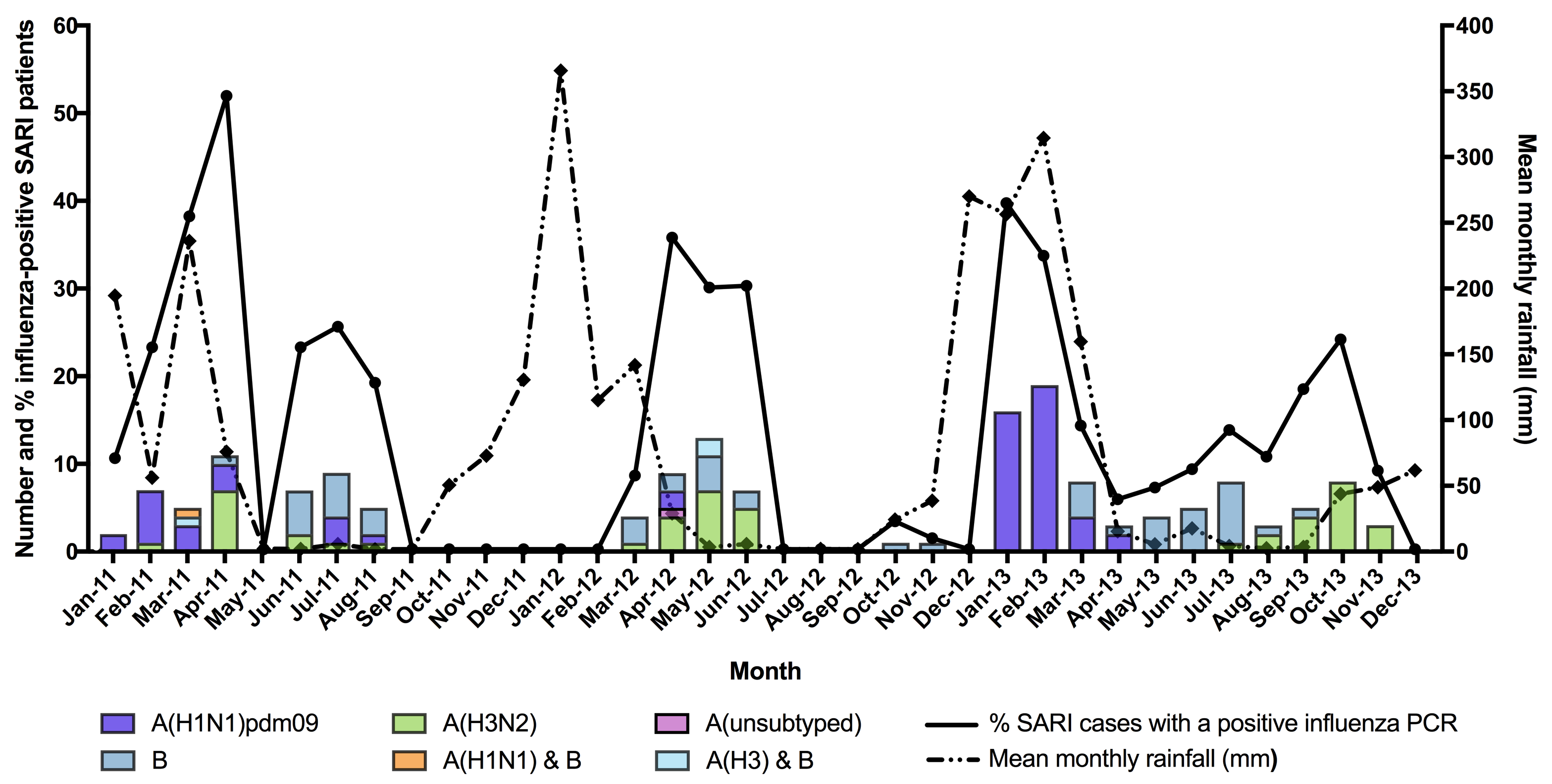

Supplement: Supplementary file 4 [file tpmd170905.SD4.tiff]
